# Volumetric imaging parameters are significant for predicting the pathological complete response of preoperative concurrent chemoradiotherapy in local advanced rectal cancer

**DOI:** 10.1093/jrr/rrz035

**Published:** 2019-06-05

**Authors:** Fengpeng Wu, Jun Wang, Congrong Yang, Chaoxi Zhou, Wenbo Niu, Jianfeng Zhang, Guanglin Wang, Yafan Yang, Guiying Wang

**Affiliations:** 1 Department of Radiation Oncology, Fourth Hospital of Hebei Medical University, 12 Jiankang Road, Shijiazhuang, Hebei, China Shijiazhuang, China; 2 Department of Colorectal Surgery, Fourth Hospital of Hebei Medical University, 12 Jiankang Road, Shijiazhuang, Hebei, China

**Keywords:** locally advanced rectal cancer, preoperative concurrent chemoradiotherapy, pathological complete response, volumetric imaging parameters, predictive values

## Abstract

Preoperative concurrent chemoradiotherapy (CCRT) as the standard treatment for locally advanced rectal cancer (LARC) has been widely used in clinic. Its efficiency influences the prognosis and the selection of subsequent treatment. The current criteria for evaluating the prognosis of patients with extremely sensitive preoperative CCRT include the clinical complete remission response (cCR) and pathological complete response (pCR), but those with cCR may not necessarily achieve pCR, and the pCR can be confirmed only after surgery. Some scholars believe that patients with pCR after CCRT can be categorized as ‘watch and wait’. Therefore, it is extremely important to find a way to predict the pCR status of patients before therapy. In this study, we examined the expression of stem cell markers and obtained direct and derivative volumetric imaging parameters before treatment. Subsequently, these factors and the general clinical data were adopted into a regression model, and the correlation between them and the pCR was analyzed. We found that the pCR of LARC was positively correlated with tumor compactness (TC), whereas it was negatively correlated with approximate tumor volume (ATV), real tumor volume (RTV), total surface area of the tumor (TSA) and tumor maximum longitudinal length (TML). In these meaningful predictors, the positive predictive values and the negative predictive values of TC were 74.73% and 94.61%, respectively. Compared with other possible predictors, TC is the most encouraging predictor of pCR. Our findings provide a way for clinicians to predict the sensitivity of preoperative CCRT and will help to select individualized treatment options for LARC patients.

## INTRODUCTION

Preoperative concurrent chemoradiotherapy (CCRT) followed by total mesorectal excision (TME) surgery, as a standard treatment for locally advanced rectal cancer (LARC), has been widely used in clinical practice, but significant differences in the response after neoadjuvant therapy exist in different patients. The criteria for evaluating the differences include clinical complete remission response (cCR) and pathological complete response (pCR), but those with cCR may not necessarily achieve pCR, and the pCR can be confirmed only after surgery. It was found that patients who achieved pCR after preoperative CCRT had a significantly better prognosis than those who did not [1]. Therefore, the approach of ‘watch and wait’ instead of surgical resection may be a better option for patients with a favorable response, particularly for those with lower rectal tumor who are unsuitable for sphincter-preserving surgery [2]. At present, there are few encouraging factors for pCR prediction in LARC patients who undergo CCRT. Therefore, it is of great significance to explore tumor response predictors.

In this study, we collected the general clinical data of 79 LARC patients, examined the expression of the CD44 spliced variant CD44v6, a tumor stem cell marker, in primary tumors, according to the results of Huh *et al.* in screening for predictors of tumor regression after preoperative CCRT for rectal cancer [3], obtained eight direct or derivative volumetric imaging parameters and analyzed the correlativity between the pCR and these factors, in order to provide guidance for clinial practice.

## MATERIALS AND METHODS

### Entry standard

This study included 79 patients with LARC who received a long course of preoperative CCRT in our hospital between May 2015 and August 2017. The eligibility criteria included: (i) histologically proven rectal adenocarcinoma; (ii) cT2N+ or cT3–4; (iii) no distant metastasis; (iv) intervals between CCRT and TME were 8–10 weeks; (v) R0-resection; (vi) availability of contrast-enhanced computed tomography (CT) for three-dimensional radiotherapy positioning; and (vii) underwent chest CT, abdominal and pelvic magnetic resonance imaging (MRI), and a transrectal ultrasound (EUS) as a part of their preoperative staging. The project was carried out in accordance with the ethical standards of the World Medical Association Declaration of Helsinki. All participants provided written informed consent for inclusion in the study. The approval was obtained from an independent ethics committee at the Fourth Hospital of Hebei Medical University (2014MEC067).

### Preoperative CCRT and surgery

All patients were administered 50.4 Gy irradiation in 1.8 Gy fractions over a 6 week period with intensity-modulated radiation therapy (IMRT) by using 6 MV photons. The targets were defined on the basis of the International Commission on Radiation Units and Measurements report no. 83 (2010) and the recommendations by Lee *et al.* in their academic writings [4]. Gross tumor volume of the primary tumor (GTV-T) and regional lymph nodes metastasis (GTV-N) were delineated using information from diagnostic MRI and EUS. The high-risk clinical target volume (CTV-H) included the GTV-T and GTV-N (if any). The low-risk clinical target volume (CTV-L) included the CTV-H, presacral, mesorectal, common iliac, internal iliac and external iliac (only in cT4 disease) lymphatic drainage area. Following that, the planning tumor volume (PTV) was enlarged 0.5–1.0 cm around the CTV-L in three-dimensional directions. The prescription doses for each patient were executed in two phases: the first phase was 45 Gy in 25 daily fractions and the dose was received by PTV; while the second phase was 5.4 Gy in three daily fractions and the dose was administered according to the CTV-H. All patients received capecitabine (825 mg m^–2^) twice a day concurrently with irradiation, and suspended drug use when radiotherapy was disrupted every weekend. Cases of severe hematological or gastrointestinal toxicity (grade 3 and higher) were not seen. Surgical resection was performed in the mesorectal plane down to the pelvic floor according to the standards of TME at ~8–10 week intervals following completion of neoadjuvant treatment. In total, 62 (78%) patients underwent low abdominal resection, and 17 (22%) underwent anterior perineal resection because the tumors were close to the sphincter. No Hartmann’s procedure was necessary in any case.

### The expression of CD44v6

CD44v6 protein in rectal primary tumor specimens obtained by colonoscopy before CCRT was analyzed by ABC immunohistochemical staining according to the manufacturer’s instructions (Abcam). The CD44v6-positive breast cancer tissue was used as a positive control. Phosphate-buffered saline (PBS; pH 7.4) instead of the primary antibody was used as the negative control. The results were evaluated independently by two experienced pathologists who did not have any knowledge of the clinical status of the specimens. The scores for CD44v6 staining were as follows: ‘–’ no staining or <10% positive cells; ‘+’ 10–20% weakly to moderately positive cells; ‘+ +’ 10–20% intensively positive cells or 20–50% weakly positive cells; and ‘+++’ 20–50% positive cells with moderate to strong reactivity or >50% positive cells [5]. In this study, the final evaluation of CD44v6 expression was described as ‘low expression’ or ‘high expression’, with low expression including ‘–’ and ‘+’ staining, and high expression including ‘+ +’ and ‘+++’ staining.

### Volumetric imaging parameters

**Fig. 1.**
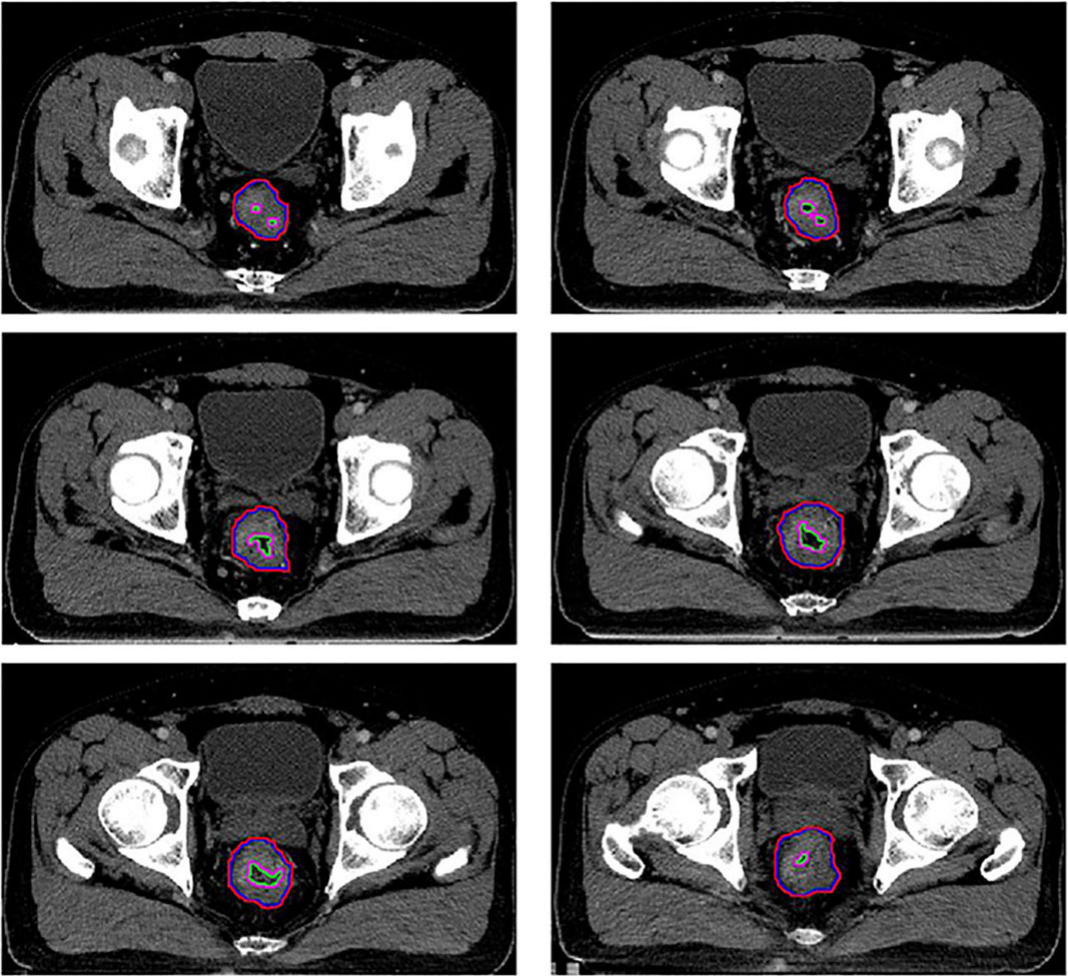
The rectal tumor of one representative patient on the axial images (contrast-enhanced CT). The red line encompasses the ATV area of every cross-sectional slice. The ATV–1 mm, the blue line, was generated from the ATV with a three-dimensional universal contraction of 1 mm length. The TSAO is the area between the red line and the blue line, encompassing the 1 mm layer volume. The green line encompasses the ITC area of every cross-sectional slice. The ITC+1 mm, the pink line, was generated from the ITC with a three-dimensional universal enlargement of 1 mm length. TSAI is the area between the pink line and the green line, encompassing the 1 mm layer volume. TSA is the sum value of TSAO and TSAI. The area between the red line and green line is the RTV. ATV, approximate tumor volume; TSAO, the tumor surface area outside the intestine; TSAI, the tumor surface area inside the intestine; TSA = TSAI + TSAO; RTV, real tumor volume.

All the enrolled patients had been subjected to pelvic high-resolution MRI and EUS 2 weeks before the CCRT. The approximate tumor volume (ATV), the volume of the rectum at the tumor position and the intestinal tube cavity (ITC) at the tumor position were described using Pinnacle version 9.1 by one radiation oncologist with >5 years experience, who took the results of MRI and EUS into account. After that, the careful examination and verification on the aforementioned roles were carried out by one senior radiotherapy physician and one experienced radiologist. The cubage values of ATV and ITC were calculated through the volume calculation function of the Pinnacle workstation. The difference values of ATV and ITC were defined as real tumor volume (RTV). We used the contraction function of Pinnacle software to generate a new parameter TSAO (the tumor surface area outside the intestine) from the ATV with a three-dimensional universal contraction of 1 mm length, then used the enlargement function of Pinnacle software to generate another new parameter TSAI (the tumor surface area inside the intestine) from the ITC with a three-dimensional universal enlargement of 1 mm length. In fact, both TSAO and TSAI were the outermost 1 mm layer volume of a tumor. The total surface area (TSA) was the sum value of TSAO and TSAI (Fig. 1). Thus, tumor compactness (TC), another parameter, was calculated from the following equation, TC = RTV/(TSA)^1.5^ [6–8]. The tumor maximum longitudinal length (TML) and the tumor maximum transverse diameter (TMD) were measured directly on the CT image.

### Pathological response evaluation

The rectal cancer regression grade (RCRG) following preoperative CCRT of rectal tumors was quantified by the data set for rectal cancer reporting of Wheeler *et al.* [9], who simplified the classification into three levels: RCRG 1, sterilization or only microscopic foci of adenocarcinoma remaining, with marked fibrosis; RCRG 2, marked fibrosis but macroscopic disease present; and RCRG 3, little or no fibrosis, with abundant macroscopic disease.

### Statistical analysis

Statistical analysis is performed using SPSS software 17.0 (SPSS, Inc., Chicago, IL, USA) and MedCalc Version 16.2. The association among these volumetric imaging parameters is analyzed by the Spearman correlation coefficients. A forward stepwise logistic regression is used to analyze the relationships between the factors and pCR. The Hosmer and Lemeshow test is used to evaluate the goodness of fit of the logistic regression model, and receiver operating characteristic (ROC) curve analysis is used to evaluate the prediction performance of the logistic regression model. In this study, *P* < 0.05 was considered statistically significant.

## RESULTS

### Patient general condition and treatment characteristics

The study population included 79 patients (46 male and 33 female), with a median age of 59.4 years (32–73.6 years), who received a long course of preoperative CCRT combined with TME. Considering the preoperative clinical tumor stage (cT) and clinical node stage (cN), 11 patients had cT2, 54 had cT3 and 14 had cT4 tumors; 11 patients had cN0, 39 had cN1 and 29 had cN2 diseases. Furthermore, the carcinoembryonic antigen (CEA) levels ranged from 1.13 to 109.91 (median 4.27) ng ml^–1^. In our cohort, 69 patients (86%) were diagnosed as non-mucinous adenocarcinoma, 3 patients (5%) as mucinous adenocarcinoma and 7 patients (9%) as adenocarcinoma with mucinous features. After TME surgery, a histopathological examination revealed 20 patients as ypT0, 14 as ypT1, 17 as ypT2, 25 as ypT3 and 3 as ypT4 tumors. The rectal tumor regression grades following preoperative CCRT were as follows: 20 (25%) cases as RCRG 1, 35 (44%) cases as RCRG 2 and 24 (30%) cases as RCRG 3. This result means that 20 patients in this study achieved pCR after preoperative CCRT.

### The expression of CD44v6

**Fig. 2.**
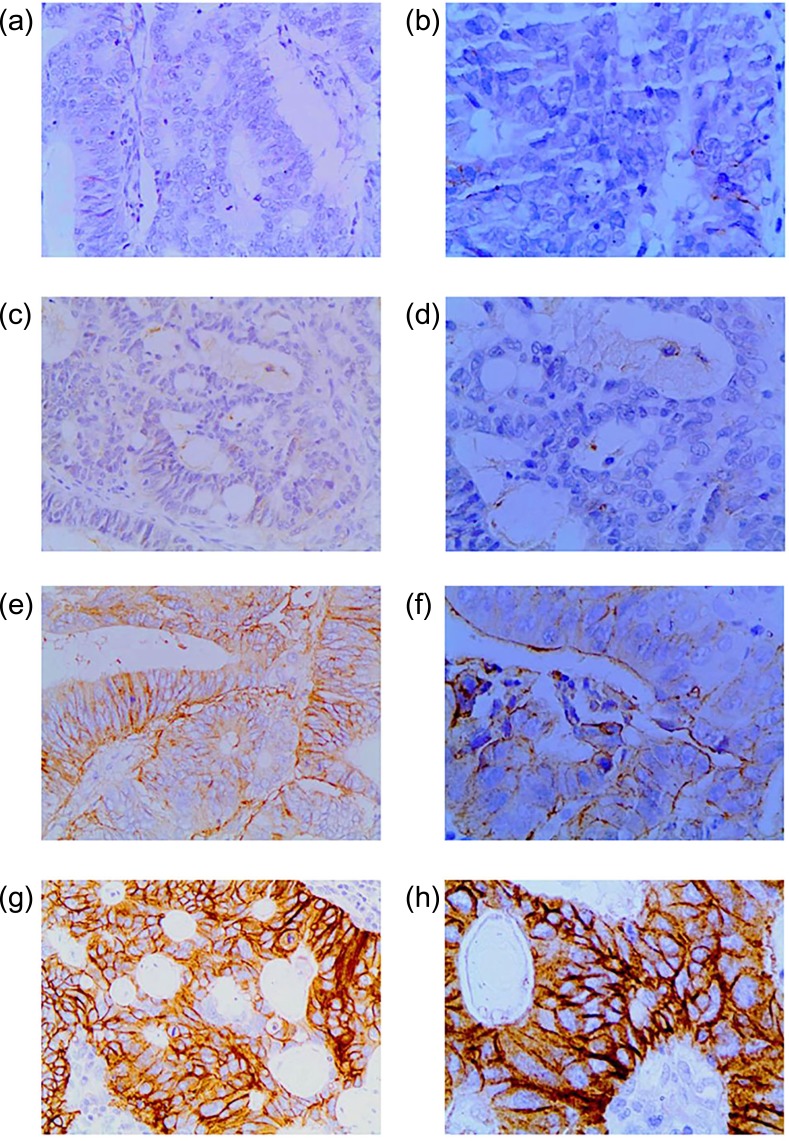
Representative photomicrographs of CD44v6 expression scoring in LARC tissues. CD44v6 is expressed in the cytoplasm. (**a**) ‘–’ expression (×200). (**b**) ‘–’ expression (×400). (**c**) ‘+’ expression (×200). (**d**) ‘+’ expression (×400). (**e**) ‘++’ expression (×200). (**f**) ‘++’ expression (×400). (**g**) ‘+++’ expression (×200). (**h**) ‘+++’ expression (×400).

CD44v6 was expressed in the cytoplasm in rectal cancer tissues. Of all 79 patients, its expression was ‘–’ in 33 cases (42%), ‘+’ in 21 cases (27%), ‘++’ in 17 cases (22%) and ‘+++’ in 8 cases (10%) (Fig. 2). In 20 patients with pCR (RCRG 1) after CCRT, 3 had high expression of CD44v6 (15%).

### Volumetric imaging parameters

We obtained the values of ATV, RTV, TSAO, TSAI, TSA, TC, TML and TMD, and analyzed the correlation among these parameters by the Spearman methods. The results revealed that there were significant positive correlations between the RTV and ATV (*r*_*s*_ = 0.99, *P* < 0.001), TML (*r*_*s*_*=* 0.53, *P* < 0.001) and TSA (*r*_*s*_*=* 0.83, *P* < 0.001), where *r*_*s*_ is the Spearman rank correlation coefficient and *P* is the probability of hypothesis testing on the Spearman rank correlation coefficient. Moreover, the association between the TC and other parameters shows that there were significant inverse correlations between the TC and TML (*r*_*s*_ = −0.30, *P* = 0.007) and TSA (*r*_*s*_ = −0.47, *P* < 0.001) (Table 1).

**Table 1.** Characteristics of tumor volumetric imaging parameters

Parameters	Median	Range	*r*_*s*_	*P-*value
ATV	52.84	14.03–269.09 cm^3^		
RTV	45.49	12.29–259.14 cm^3^		
TSAO	9.07	1.77–36.63 cm^2^		
TSAI	2.15	0.09–11.02 cm^2^		
TSA	11.30	2.67–41.56 cm^2^		
TC	1.21	0.51–4.55		
TML	8.30	3.50–16.30 cm		
TMD	4.50	2.51–8.13 cm		
**Correlation of tumor volumetric imaging parameters**				
ATV vs RTV			0.99	<0.001
TML vs RTV			0.53	<0.001
TMD vs RTV			0.03	0.79
TSA vs RTV			0.83	<0.001
TML vs TC			−0.30	0.007
TMD vs TC			−0.11	0.334
TSA vs TC			−0.47	<0.001
ATV vs TC			−0.02	0.88
RTV vs TC			−0.04	0.743

ATV = approximate tumor volume; RTV = real tumor volume; TSAO = tumor surface area outside the intestine; TSAI = tumor surface area inside the intestine; TSA = total surface area of tumor; TC = tumor compactness; TML = tumor maximum longitudinal length; TMD = tumor maximum transverse diameter; *r*_*s*_*=* Spearman rank correlation coefficient; *P*-value = the probability value of hypothesis testing of the Spearman rank correlation coefficient.

### Univariate analysis for predictors of pCR

Fifteen possible predictors of pCR were incorporated into the evaluation system one by one. Each factor was assigned and stratified according to its characteristics (Table 2). We found that age, gender, clinical tumor stage, clinical node stage, pathological histology and serum CEA levels were not associated with the pCR. In contrast to clinical parameters, we discovered that among various possible tumor volumetric predictors, TC was significantly and positively correlated with rectal pCR [odds ratio (OR) = 3.11, *P* < 0.001], whereas TML, ATV, RTV and TSA were significantly and negatively correlated with rectal pCR. The expression of CD44v6 was marginally negatively associated with the pCR (OR = 0.30, *P* = 0.075) where *P* indicates the probability of factors to pCR status under univariate analysis (Table 3).

**Table 2.** Fifteen possible predictors of pCR and its substitute and layering

Factors	Category and the definition
Age (years)	≤40 = 1; 41–50 = 2; 51–60 = 3; 61–70 = 4; ≥71 = 5
Gender	Male = 1; female = 2
Clinical tumor stage	T2 = 1; T3 = 2; T4 = 3
Clinical node stage	N0 = 0; N1 = 1; N2 = 2
Pathological histology	Non-mucinous adenocarcinoma = 0; Mucinous adenocarcinoma and adenocarcinoma with mucinous feature = 1
CEA (ng ml^–1^)	≤5 = 1; >5 = 2
CD44v6	low expression = 0, high expression = 1
TML (cm)	≤5 = 1; >5 and ≤8 = 2; >8 and ≤11 = 3; >11 = 4
TMD (cm)	≤3 = 1; >3 and ≤5 = 2; >5 and ≤7 = 3;>7 = 4
ATV (cm^3^)	≤35 = 1; >35 and ≤70 = 2; >70 and ≤105 = 3; >105 and ≤140 = 4;>140 = 5
RTV (cm^3^)	≤35 = 1; >35and ≤70 = 2; >70 and ≤105 = 3; >105 and ≤140 = 4;>140 = 5
TSAI (cm^2^)	≤2 = 1; >2 and ≤4 = 2; >4 and &≤6 = 3;>6 = 4
TSAO (cm^2^)	≤10 = 1; >10&≤20 = 2; >20&≤30 = 3;>30 = 4
TSA (cm^2^)	≤10 = 1; >10 and ≤20 = 2; >20 and ≤30 = 3; >30 = 4
TC	≤1 = 1; >1.0 and ≤1.5 = 2; >1.5and ≤2 = 3;>2 and ≤2.5 = 4; >2.5 = 5

ATV = the approximate tumor volume; RTV = the real tumor volume; TSAO = the tumor surface area outside the intestine; TSAI = the tumor surface area inside the intestine; TSA = the total surface area of the tumor; TML = the tumor maximum longitudinal length; TMD = the tumor maximum transverse diameter; TC = tumor compactness

**Table 3.** Univariate analysis for possible predictors of pCR for preoperative CCRT in LARC

Factors	Univariate analysis		
	OR	*P*-value	95% CI
Age	1.32	0.3	0.78–2.23
Gender	1.57	0.39	0.56–4.34
cT	1.05	0.912	0.43–2.60
cN	0.80	0.551	0.38–1.68
Histology	0.57	0.36	0.17–1.89
CEA	0.74	0.561	0.27–2.05
CD44v6	0.30	0.075	0.08–1.13
TML	0.35	0.007	0.16–0.75
TMD	0.82	0.612	0.38–1.78
ATV	0.40	0.011	0.20–0.81
RTV	0.40	0.015	0.19–0.83
TSA	0.18	0.002	0.06–0.53
TSAO	1.21	0.575	0.63–2.32
TSAI	1.54	0.126	0.89–2.66
TC	3.11	<0.001	1.66–5.84

cT = clinical tumor stage; cN = clinical node stage; CEA = carcinoembryonic antigen; ATV = approximate tumor volume; RTV = real tumor volume; TSAO = tumor surface area outside the intestine; TSAI = tumor surface area inside the intestine; TSA = total surface area of the tumor; TC = tumor compactness; TML = tumor maximum longitudinal length; TMD = tumor maximum transverse diameter; OR = odds ratio; CI = confidence interval.

### Multivariate analysis for predictors of pCR

According to the results of the correlation analysis listed in Table 1, we found that there was a certain degree of correlation among these volumetric imaging parameters. In order to reduce the distortion of model evaluation, we conducted collinearity statistics for all selected factors before performing the multivariate logistic regression analysis. A variance inflation factor (VIF) of predictors ≥10, including RTV and ATV, was thought to be highly correlated with at least one of the other predictors in the aforementioned model. When excluding ATV or RTV from the model, we observed that the VIF of all factors was <7. In this study, we chose the factors of age, gender, clinical tumor stage, clinical node stage, pathological histology, serum CEA levels and the expression of CD44v6, TML, TMD, ATV, RTV, TSAI, TSAO, TSA and TC for multivariate analysis. When we excluded ATV or RTV from the regression model, the significance of the Hosmer–Lemeshow test goodness of fit was 0.40 and 0.14, respectively, indicating that the logistic regression model fit the data well. Multivariate analysis showed that TC was a positive predictor of pCR (*P* < 0.001), RTV and ATV were negative predictors; *P-*values were 0.01 and 0.007, respectively (Table 4). Logistic regression models were $Z_{1}=\frac{e^{-1.705-1.298\times RTV+1.2999\times TC}}{1+e^{-1.705-1.298\times RTV+1.2999\times TC}}$ and $Z_{2}=\frac{e^{-1.620-1.235\times ATV+1.293\times TC}}{1+e^{-1.620-1.235\times ATV+1.293\times TC}}$ In the above two models, *Z*_1_ represents the probability of patients obtaining pCR when RTV is included in the independent variables, and *Z*_2_ represents the probability of patients obtaining pCR when ATV is included in the independent variables. We next evaluated the predictive performance of the two logistic regression models by calculating the area under the curve (AUC) of the ROC curve. The AUC was 0.846 and 0.853, respectively, which indicated that both models have good prediction value and, when the values of *Z*_1_ or *Z*_2_ are greater than their respective optimal cut-off values, LARC patients can be considered to be able to obtain pCR after preoperative CCRT.

**Table 4.** Significant predictors of pCR for preoperative CCRT in LARC

Factors	Multivariate analysis		
	OR	*P*-value	95% CI
RTV	0.273	0.010	0.102–0.732
TC	3.667	0.000	1.792–7.502
Constant	0.182	0.096	
ATV	0.291	0.007	0.118–0.716
TC	3.643	0.000	1.783–7.444
Constant	0.198	0.113	

RTV = real tumor volume; TC = tumor compactness; ATV = approximate tumor volume; OR = odds ratio; *P*-value = the probability of factors to pCR status under multivariate analysis conditions; CI = confidence interval.

### Evaluation of predictive value

**Fig. 3.**
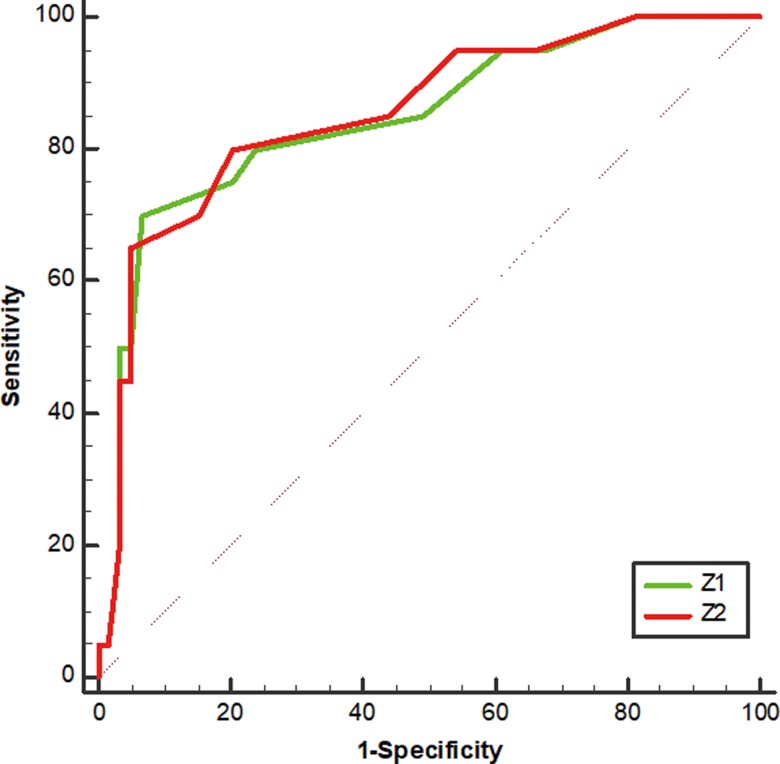
ROC curve of logistic regression equations in LARC.

**Fig. 4.**
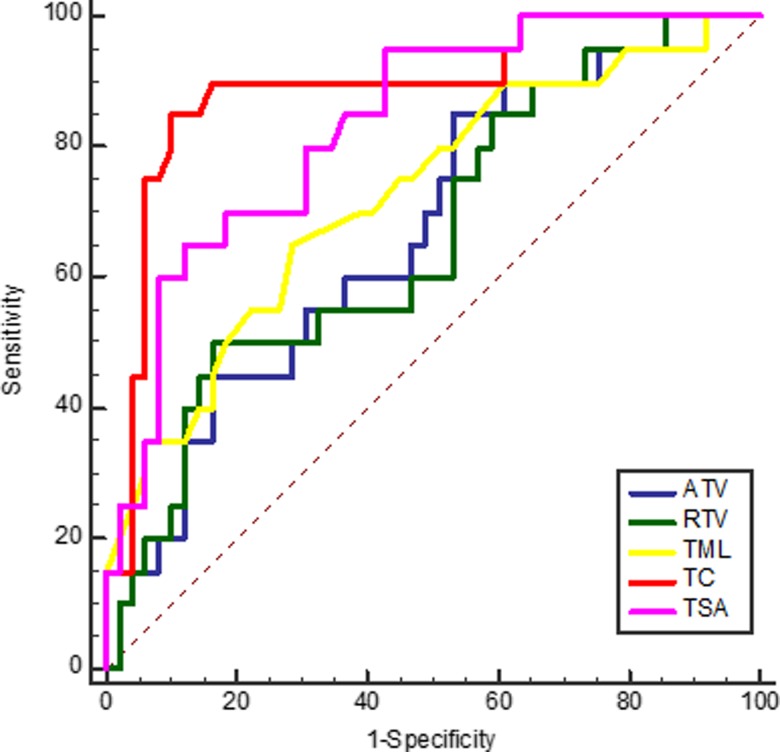
ROC curve of ATV, RTV, TML, TC and TSA using the pCR as test variable.

To determine the predictive value of the above logistic regression equations and the performance of volumetric imaging parameters significantly associated with the pCR prediction of LARC, ROC analysis was conducted. The results indicated that on the basis of the optimal cut-off values of 0.40, 0.43, 56.06 cm^3^, 55.88 cm^3^, 7.5 cm, 1.4 and 7.9 cm^2^, the sensitivity of Z_1_, *Z*_2_, ATV, RTV, TML, TC and TSA was 70.00, 65.00, 85.00, 85.00, 65.00, 85.00 and 60.00%, the specificity was 93.22, 94.92, 47.46, 42.37, 69.49, 89.83 and 91.53%, the positive predictive values were 77.78, 81.27, 35.57, 33.48, 42.09, 74.73 and 70.73%, and the negative predictive values were 90.16, 88.89, 90.27, 89.22, 85.33, 94.61 and 87.02%, respectively(Fig. 3, Fig. 4).

### Correlation analysis for pCR prediction in the non-mucinous adenocarcinoma subgroup

After excluding the three patients with mucinous adenocarcinoma and seven patients with adenocarcinoma with mucinous feature, we found that ATV, RTV, TML, TC and TSA were significantly correlated with the sensitivity of CCRT in 69 non-mucinous adenocarcinoma patients in univariate analysis. In multivariate analysis, when we excluded ATV or RTV from the regression model, the significance of the Hosmer–Lemeshow test goodness of fit was 0.39 and 0.79, respectively, indicating that the logistic regression model fit the data well. Multivariate analysis showed that TC was a positive predictor of pCR (*P* = 0.001), RTV and ATV were negative predictors; *P-*values were 0.017 and 0.01, respectively (Table 5). Logistic regression equations were $Z_{3}=\frac{e^{-1.429-1.227\times RTV+1.164\times TC}}{1+e^{-1.429-1.227\times RTV+1.164\times TC}}$ and $Z_{4}=\frac{e^{-1.323-1.19\times ATV+1.147\times TC}}{1+e^{-1.323-1.19\times ATV+1.147\times TC}}$. In the above two models, *Z*_3_ represents the probability of patients obtaining pCR when RTV is included in the independent variables, and *Z*_4_ represents the probability of patients obtaining pCR when ATV is included in the independent variables. The AUC was 0.822 and 0.826, respectively, which indicated that both models have good prediction and, when the values of *Z*_3_ or *Z*_4_ are greater than their respective optimal cut-off values, rectal non-mucinous adenocarcinoma patients can be considered to be able to obtain pCR after preoperative CCRT.

**Table 5.** Significant predictors of pCR for preoperative CCRT in locally advanced rectal non-mucinous adenocarcinoma

Factors	Multivariate analysis		
	OR	*P-*value	95% CI
RTV	0.293	0.017	0.107–0.802
TC	3.203	0.001	1.575–6.515
Constant	0.240	0.162	
ATV	0.304	0.01	0.123–0.754
TC	3.148	0.001	1.559–6.357
Constant	0.266	0.193	

RTV = real tumor volume; TC = tumor compactness; ATV = approximate tumor volume; OR = odds ratio; CI = confidence interval; *P*-value = the probability of factors to pCR status under multivariate analysis condition.

### Evaluation of predictive value for non-mucinous adenocarcinoma subgroup

**Fig. 5.**
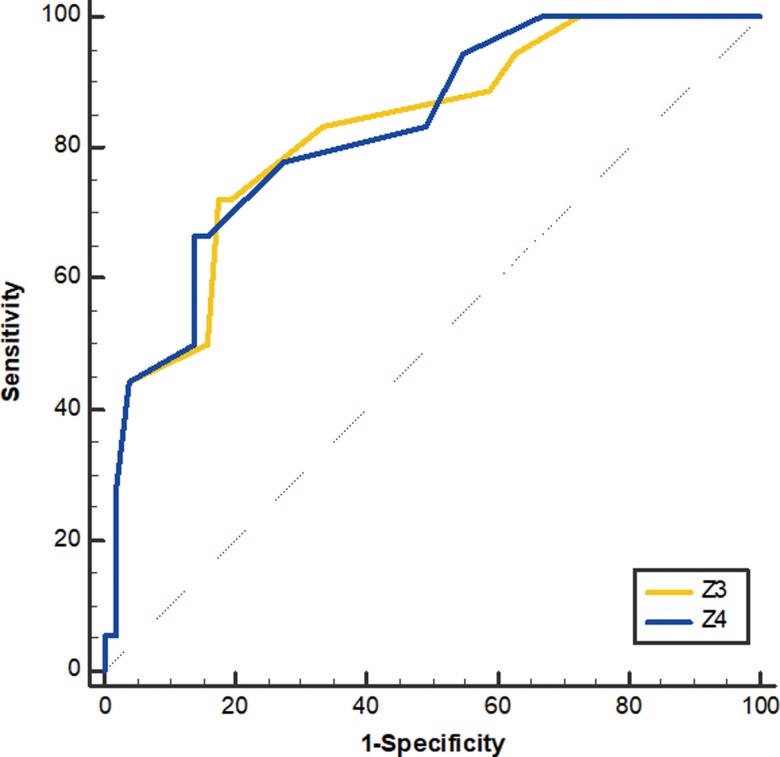
ROC curve of logistic regression equations in the non-mucinous adenocarcinoma subgroup.

**Fig. 6.**
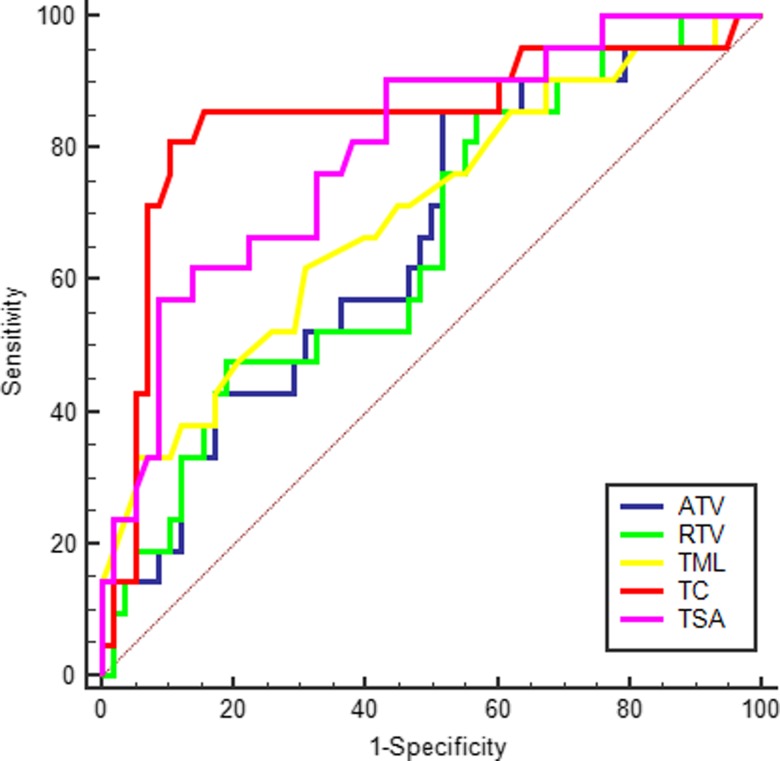
ROC curve of ATV, RTV, TML, TC and TSA using the pCR as test variable in the non-mucinous adenocarcinoma subgroup.

ROC analysis of the rectal non-mucinous adenocarcinoma indicated that on the basis of the optimal cut-off values of 0.39, 0.42, 56.06 cm^3^, 30.84 cm^3^, 7.5 cm, 1.4 and 8.36 cm^2^, the sensitivity of *Z*_3_, *Z*_4_, ATV, RTV, TML, TC and TSA was 72.22, 66.67, 85.00, 50.00, 65.00, 85.00 and 65.00%, the specificity was 82.35, 86.27, 46.94, 83.67, 71.43, 89.80 and 87.76%, the positive predictive values were 59.08, 66.67, 35.34, 51.09, 43.70, 73.98 and 64.44%, and the negative predictive values were 89.35, 88.00, 90.17, 83.06, 85.68,94.61 and 88.0%, respectively (Fig. 5, Fig. 6).

## DISCUSSION

We studied the correlation between the volume-related parameters of LARC and the pCR after preoperative CCRT. Univariate analysis showed that TML, ATV, RTV and TSA could be used as positive predictors. In order to reduce the probability of biased estimates and inflated standard errors among the various predictors in the multivariate analysis, we excluded the possible risk factors from the regression model. Multivariate analysis showed that both ATV and RTV could be used as the independent predictors of pCR in patients with LARC after neoadjuvant treatment. These results were consistent with the study of Hsu *et al.* [6].

Several studies had shown that the tumor size was usually negatively correlated with its radiation sensitivity [10–14], which might be due to the following reasons. (i) As is known, due to the spatial and temporal heterogeneity of causes of tumors, both the mutation rate and the expression patterns of driver genes increase gradually as the tumor volume increases. (ii) During the process of tumor evolution, the three-dimensional structures of cancer cells and the extracellular matrix were in a dynamic process, which led to the increase of tumor spatial heterogeneity, the oxygenation status of tumor cells, the nutrient supply level of the tumor and the altered expression of growth factors [15–20]. (iii) Radiation therapy can induce ane increase of reactive oxygen species (ROS) in the mitochondria of tumor cells and then lead to a second killing of cancer cells. It was found that the activity and expression level of the ROS clearance factor superoxide dismutase (SOD) in cancer tissue was positively correlated with the degree of malignancy of the lesion [21–23]. Therefore, we speculate that with the progress and the volume increase of the tumor, the ability of SOD to scavenge ROS is enhanced, which makes cancer cells more resistant to radiation.

Because rectal cancer is a neoplasm located in hollow organs, in the process of radiotherapy target delineating and radiotherapy implementing, the intestinal cavity at the same level as the tumor is difficult to exclude from GTV. Hence, the ATV in our study is actually equivalent to the GTV. Obtaining the RTV value usually requires consulting the MRI image. Although it is more accurate than ATV in describing the size of the tumor burden before treatment, the acquisition of RTV is more complicated. Therefore, it is more convenient for radiation therapists to use ATV than RTV when predicting the pCR of LARC before neoadjuvant therapy.

In order to evaluate the prediction performance of volumetric imaging parameters significantly associated with the pCR, ROC curve analysis was employed. The results showed that the positive predictive values and negative predictive values of RTV and ATV for pCR prediction were 33.48, 89.22, 35.57 and 90.27%, respectively. This means that the advantages of ATV and RTV in predicting the response of LARC to preoperative CCRT are mainly manifested in the screening of non-pCR in patients who are not sensitive. We also conducted ROC analysis on the predictive value of logistic regression equations and found that the AUC values of all regression models we obtained are >0.8, which means that these equations have good predictive value. When the *Z* value is greater than the corresponding optimal cut-off values, these equations can be used to predict the sensitivity of patients to preoperative CCRT.

There was no correlation between the tumor maximum transverse diameter (TMD) and the status of pCR in our study, which is inconsistent with the findings of Janjan *et al.* [24]. In that study, the tumor regression rate was >73% in patients with a maximum tumor diameter of <5 cm. The reason may be that the endpoint of our study was pCR, while that of Janjan *et al.* was the reduction of clinical stage. In addition, we found that it is difficult to completely exclude the intracavity volume surrounded by the tumor tissue from the measurement range when measuring TMD, which could result in deviation of the measured values.

TC is a parameter originating from tumor-measurable volumetric imaging parameters and has been found to be closely related to tumor morphology and invasiveness [7, 25, 26]. According to Fave *et al.* [27], TC not only reflects the information on tumor volume and TNM, but also the prognosis of non-small cell lung cancer patients. From the equation of TC, there seems to be an inverse relationship between TC and TSA. It is well known that TSA determines the degree of tumor contact with the surrounding tissues and organs. A study by Agner *et al.* revealed that the TC value of triple-negative breast cancer was significantly higher than that of HER2-positive breast cancer, suggesting that the edge of triple-negative breast cancer is smoother than that of HER2-positive breast cancer [8]. Therefore, they hypothesized that the correlation between TC and tumor invasiveness may be due to the size of the TSA. Our opinion is similar to the view of Frieboes *et al.* [25], who suggested that the mechanical force of tumor bearing and its three-dimensional diffusion gradient affect TC.

The relationship between TC and tumor biological characteristics is not yet clear. Some researchers believe that the β-catenin-related signaling pathway affects the adhesion between tumor cells and then affects the TC [28]. Another recent study revealed that down-regulation of 45A non-coding RNA (ncRNA) increased the compactness of tumor nodules in a mouse model of subcutaneous neuroblastoma, suggesting that its regulation might influence the prognosis of patients [29].

In this study, the evaluation of positive lymph nodes was executed based on the image data. Several studies had found that neither CT nor MRI could accurately identify malignant pelvic lymph nodes [30, 31]. Instead of calculating the RTV, TSA and TC of lymph nodes, we evaluated the N staging according to the number of possible positive lymph nodes. In addition, in establishing a multivariate model, CEA was stratified on the basis of clinically recommended reference values (5 ng ml^–1^), while the group with CEA >5 ng ml^–1^ was not further stratified. All of these are likely to cause research bias, which may also be a limitation of our study.

In order to further clarify the relationship between candidate factors and the pCR of non-mucinous adenocarcinoma patients after CCRT, we excluded three cases with mucinous adenocarcinoma and seven cases with adenocarcinoma with mucinous features from 79 patients enrolled for further analysis. The results showed that TC, RTV and ATV could still be used as independent predictors to evaluate the patient’s pCR status. The predictive advantage of ATV was its sensitivity, whereas the predictive power of RTV was its specificity. We thought that the occurrence of this condition may be due to the fact that the RTV of early rectal adenocarcinoma is smaller, and the intestinal tube cavity in the ATV range is larger.

An increasing number of studies have found a synergistic relationship between immunotherapy and radiotherapy for tumors. The study of 46 patients with soft tissue sarcoma who received preoperative radiotherapy by Patel *et al.* found that radiotherapy could enhance the expression of PDL-1 in tumor-associated macrophages [32]. Zhuang *et al.* found that PD-1 inhibitor not only can enhance the curative effect of tumor in the radiation field, but also has a distant therapeutic effect on the tumor [33]. In addition to PD-I and PDL-1, commonly used immunoassays for cancer therapy include microsatellite instability (MSI) and tumor mutational burden (TMB). Hasan *et al.* obtained data from the National Cancer Database (NCDB) and analyzed the relationship between pCR status and MSI in 5086 rectal cancer patients who received preoperative CCRT, and found that MSI(+) was related to a low pCR rate after CCRT [34]. The TMB status and chemotherapy efficacy of patients with colorectal cancer were analyzed by Pai *et al.*, who found that the TMB level was not correlated with progression-free survival (PFS) of patients receiving oxaliplatin-based chemotherapy, while patients receiving irinitecan-based chemotherapy in TMB-L had more advantages in terms of PFS [35]. At present, the relationship between TMB and preoperative CCRT of rectal cancer, as well as the relationship between various tumor immunological indicators and tumor volume imaging parameters have not been reported. In our study, only one patient had immunotherapy detection before CCRT, and we will continue to accumulate relevant data in subsequent studies.

In summary, we found that TC, RTV and ATV, as volumetric parameters, are of great significance for predicting pCR in neoadjuvant therapy patients, and the predictive value of TC was the highest among all parameters. We hold the opinion that TC plays an important role in CCRT response and can guide the selection of subsequent treatment strategies. At present, the mechanism of the relationship between tumor volume-related parameters and chemoradiotherapy sensitivity is not clear, but it might open up new ideas for our further research.
